# Identification of novel radiation-induced p53-dependent transcripts extensively regulated during mouse brain development

**DOI:** 10.1242/bio.20149969

**Published:** 2015-02-13

**Authors:** Roel Quintens, Tine Verreet, Ann Janssen, Mieke Neefs, Liselotte Leysen, Arlette Michaux, Mieke Verslegers, Nada Samari, Giuseppe Pani, Joris Verheyde, Sarah Baatout, Mohammed A. Benotmane

**Affiliations:** 1 Radiobiology Unit, Belgian Nuclear Research Centre, SCK•CEN, B-2400 Mol, Belgium; 2 Laboratory of Neural Circuit Development and Regeneration, Animal Physiology and Neurobiology Section, Department of Biology, KU Leuven, B-3000 Leuven, Belgium; 3 Cell Systems and Imaging Research Group (CSI), Department of Molecular Biotechnology, Ghent University, B-9000 Ghent, Belgium; ‡ Present address: Nutritional Biochemistry and Space Biology Lab, Department of Pharmacology and Bio-molecular Sciences, Università degli Studi di Milano, 20122 Milano, Italy.

**Keywords:** Alternative splicing, Development, Embryonic brain, Ionizing radiation, Neuronal differentiation, p53 targets

## Abstract

Ionizing radiation is a potent activator of the tumor suppressor gene p53, which itself regulates the transcription of genes involved in canonical pathways such as the cell cycle, DNA repair and apoptosis as well as other biological processes like metabolism, autophagy, differentiation and development. In this study, we performed a meta-analysis on gene expression data from different *in vivo* and *in vitro* experiments to identify a signature of early radiation-responsive genes which were predicted to be predominantly regulated by p53. Moreover, we found that several genes expressed different transcript isoforms after irradiation in a p53-dependent manner. Among this gene signature, we identified novel p53 targets, some of which have not yet been functionally characterized. Surprisingly, in contrast to genes from the canonical p53-regulated pathways, our gene signature was found to be highly enriched during embryonic and post-natal brain development and during *in vitro* neuronal differentiation. Furthermore, we could show that for a number of genes, radiation-responsive transcript variants were upregulated during development and differentiation, while radiation non-responsive variants were not. This suggests that radiation exposure of the developing brain and immature cortical neurons results in the p53-mediated activation of a neuronal differentiation program. Overall, our results further increase the knowledge of the radiation-induced p53 network of the embryonic brain and provide more evidence concerning the importance of p53 and its transcriptional targets during mouse brain development.

## INTRODUCTION

The tumor suppressor protein p53 is indisputably one of the central players of cancer biology as over half of all human cancers carry inactivating mutations in the *TP53* gene (Soussi et al., 2005). It is therefore no wonder that *TP53* (*Trp53* in the mouse) has become one of the most intensively investigated genes since it was first discovered more than three decades ago (DeLeo et al., 1979). One of its first identified functions was that of a DNA binding transcription factor (Kern et al., 1991) which activates or suppresses genes, mostly those involved in cell cycle arrest, DNA repair, apoptosis and senescence as a response to various cellular stresses including DNA damage. This is probably the most classic function of p53, which serves to stall the cell cycle to allow cells to repair the DNA before the cycle can be resumed. However, when the damage is too severe to be properly repaired, apoptosis, senescence or, in the case of embryonic stem cells, premature differentiation (Lin et al., 2005) may be induced, safeguarding the organism from developing neoplasia (Vousden and Prives, 2009). Although the transcription-dependent functions of p53 seem to be most important – 90% of cancer-related p53 mutations occur within the DNA binding domain (Soussi et al., 2005) – p53 can also induce apoptosis independently of its role as a transcription factor. In this case, p53 protein translocates to the mitochondria and triggers apoptosis via activation of pro-apoptotic Bcl-2 family members. This way of inducing cell death can occur very fast (within 30 min) and can precede the induction of pro-apoptotic p53 target genes (Erster et al., 2004). Exactly how p53 regulates different cell fates in response to DNA damage is not yet fully understood, but it has been shown to be dependent on the cell type, cell cycle phase, as well as the dynamics of p53 activation. DNA damage induced by γ-radiation initiates pulses in p53 protein levels of which the number, but not the amplitude or frequency depend on the dose (Batchelor et al., 2011). Recently, it was demonstrated that the fate of γ-irradiated cells changes by additional treatment with the Mdm2 inhibitor Nutlin-3 resulting in a sustained induction of p53 levels (Purvis et al., 2012). Although these canonical functions have long been associated with the tumor suppression activity of p53, two recent studies showed that the combined loss of p53-dependent cell cycle arrest, apoptosis and senescence in p53 mutants is not sufficient to abrogate its effect on tumor suppression (Brady et al., 2011; Li et al., 2012b). This suggests that other p53-mediated mechanisms, such as glycolysis and the regulation of oxidative stress may be critical components for tumor suppression (Li et al., 2012b).

Indeed, in recent years, p53 itself and a number of its transcriptional targets have been shown to also play a role in other biological processes such as energy metabolism, angiogenesis, autophagy, immunity, cellular differentiation, cell motility and migration, cell-cell communication and (neural) development (Menendez et al., 2009; Riley et al., 2008; Vousden and Prives, 2009). The latter can easily be envisaged by the regulatory role of p53 in eliciting physiological neuronal apoptosis during brain development, which is necessary for the correct sculpting and wiring of the brain. Several studies, however, have also demonstrated that p53 is involved in neuronal differentiation, axon guidance, neurite outgrowth and axonal regeneration (Di Giovanni and Rathore, 2012). For instance, p53 has been shown to promote neurite outgrowth in both PC12 cells and primary neurons through a mechanism depending on CBP/p300 and P/CAF-mediated acetylation of p53 leading to subsequent transcriptional activation of neurite outgrowth promoting genes such as GAP-43 and Coronin 1b (Gaub et al., 2010; Tedeschi et al., 2009). The importance of p53 for normal brain development is further exemplified by the observation that 16% of surviving p53^−/−^ mice develop exencephaly, most probably as a result of either cellular overgrowth or reduced apoptosis in the brain (Tedeschi and Di Giovanni, 2009). The lack of complete penetrance of this phenotype, suggests that the absence of p53 in these mice can be partially compensated for by other genes, for instance by the p53 family members p63 and p73, both of which have been shown to play important roles during neural development (Quadrato and Di Giovanni, 2012). Furthermore, also inappropriate activation of the p53 pathway during embryonic development has been shown to result in neural tube defects (Van Nostrand et al., 2014) or microcephaly-like brain defects when this activation is restricted to the embryonic cerebral cortex (Pollock et al., 2014).

Nevertheless, conflicting results exist regarding the role of p53 in neuronal differentiation. For instance, mouse neuroblasts either coming from p53^−/−^ mice or treated with p53 antisense oligonucleotides displayed accelerated neuronal differentiation (Ferreira and Kosik, 1996). Moreover, a recent study showed that loss of p53 function in neural stem cells leads to enhanced proliferation and accelerated differentiation. At the level of the brain, this is reflected by an increase in neurogenesis at the expense of gliogenesis during embryonic development in p53^−/−^ mice (Liu et al., 2013). In neural progenitors on the other hand, the loss of p53 function results in early neurogenesis, which could be partially reversed by restoring its function or treatment with antioxidants (Forsberg et al., 2013). Together, these data demonstrate that the role of p53 in the developing brain is highly cell type-dependent.

A complete understanding of the exact roles of p53 is further hindered by the shear complexity of its regulation. For instance, up to ten different p53 isoforms have been identified so far (due to alternative splicing, promoter usage or translational initiation sites) and each of these can be modified by several post-translational modifications (phosphorylation, acetylation, ubiquitination, etc.) (Hollstein and Hainaut, 2010; Kruse and Gu, 2009). Moreover, in different cell types p53 regulates different target genes, some of which are also known to express different isoforms with sometimes opposing functions (e.g. pro- or anti-apoptotic). This explains why after more than three decades of intensive research, many questions concerning the different roles of this important protein remain unanswered.

In this study, we set out to identify genes and transcript variants that were altered at an early stage after radiation exposure of the developing mouse brain or immature primary cortical neurons, an *in vitro* model of early differentiating neurons. Although gene ontology enrichment analysis showed that this signature was enriched in classical DNA damage response pathways such as apoptosis and cell cycle arrest, this enrichment was based on only a fraction of the genes. Other genes from the signature were either poorly characterized or not enriched in specific biological functions. However, prediction of transcription factor regulation indicated that almost all of these genes were targets of p53. We therefore validated several of the genes from our signature as being novel genuine p53 targets using quantitative reverse transcriptase PCR (qRT-PCR) and chromatin immunoprecipitation (ChIP). Furthermore, we found that almost all of these genes are significantly enriched during normal embryonic brain development. This is in contrast to the majority of genes that are involved in cell cycle regulation and DNA repair, including p53 itself, which are normally repressed during brain development. This suggests that these radiation-responsive genes mediate important brain-related functions independent of their potential role in the DNA damage response which is further exemplified by our observation that they are highly regulated in mouse models of Huntington disease (HD) and microcephaly. Together, our data provide new insight into the p53 transcriptional network in the developing mouse brain as well as in some of the transcriptional changes that occur during the earliest stages of mouse brain development.

## MATERIALS AND METHODS

### Animals

All animal experiments were handled in agreement with the Belgian laboratory animal legislation and approved by the local SCK-CEN/VITO ethical committees (ref. 02-012). C57BL/6J and Balb/cJ@Rj wild type (Janvier/Bio-services) and *Trp53* heterozygous (p53^+/−^) mice in C57BL/6J background (in-house breeding) were maintained in a normal 12∶00/12∶00 light/dark cycle. To minimize differences in time of fertilization, mating of mice occurred between 07:30 am to 09:30 am. For p53 transgenic mice, tail DNA was used for *Trp53* locus genotyping by PCR. To eliminate the possible influence of gender on differential gene expression, microarray hybridizations were performed using only male embryos for all conditions. For all experiments, mice from at least three different litters were used as biological replicates to further rule out possible differences related to the developmental stage of the embryos.

### Primary cortical neuron cell cultures

Primary cortical neuron cultures were prepared from C57BL6/J (for qRT-PCR) or BALB/cJ@Rj (for microarray experiments) mouse embryos, as previously described (Samari et al., 2013). For long-term cultures, half of the medium was refreshed every two to three days starting on the fifth day *in vitro*.

### Primary astroglia cell cultures

Cortical astroglia cell cultures were prepared as described (Kaech and Banker, 2006) using 1-day old mouse pups.

### X-irradiation

At E11 or E14, pregnant females were whole body irradiated with different doses (E11: 0.1, 0.2, 0.5 or 1.0 Gy; E14: 0.2 or 0.5 Gy) at a dose rate of 0.35 Gy/min using a Pantak RX, 250 kV–15 mA, 1 mm Cu filter installation. Calibration of the X-ray tube was performed using an ionization chamber measuring air kerma. Sham-exposed mice were used as controls. At 2 h after the irradiation, mice were sacrificed by cervical dislocation, embryos were isolated and either the whole brains (E11) or the separated cortices and hippocampi (E14) were microdissected and snap-frozen in liquid nitrogen until further manipulations.

Primary cortical neuron cultures were grown for 14 h (microarrays) or for one to seven days (qRT-PCR) prior to X-irradiation using a similar instrumental set-up as for the animals. Cells were irradiated with doses of 0.2 or 0.5 Gy (microarrays), 1.0 Gy (qRT-PCR) or sham-irradiated. For experiments using the p53 transcriptional inhibitor α-pifithrin (α-PFT; P4236, Sigma-Aldrich, Diegem, Belgium), cells were treated with either 10 µM α-PFT or 1% DMSO 90 min prior to the irradiation. RNA was extracted at 2 h or 6 h post-irradiation.

### Microarray preparation and analysis

Total RNA was extracted from flash frozen tissues or cells using the AllPrep DNA/RNA/protein Mini kit (Qiagen, Hilden, Germany), quality-controlled using the 2100 BioAnalyzer (Agilent, Santa Clara, CA, USA) and quantified using the Nanodrop 2000c spectrophotometer (Thermo Fisher Scientific, Waltham, MA, USA). Only samples with a RIN >8 were used for hybridization for 16 h at 45°C onto Affymetrix Mouse Gene 1.0 ST arrays (Affymetrix, Santa Clara, CA, USA). Arrays were washed and stained using the GeneChip Hybridization, Wash and Stain kit (Stain Module) (Affymetrix) and scanned using an Affymetrix GCS3000 scanner.

For microarray hybridization only male embryos were used and each condition was performed on at least three biological replicates from different litters except for E16 embryos for which two biological replicates were used. All microarray data are available in MIAME compliant format at the ArrayExpress database under accession codes E-MTAB-2622 and E-MTAB-2632.

**Fig. 1.**
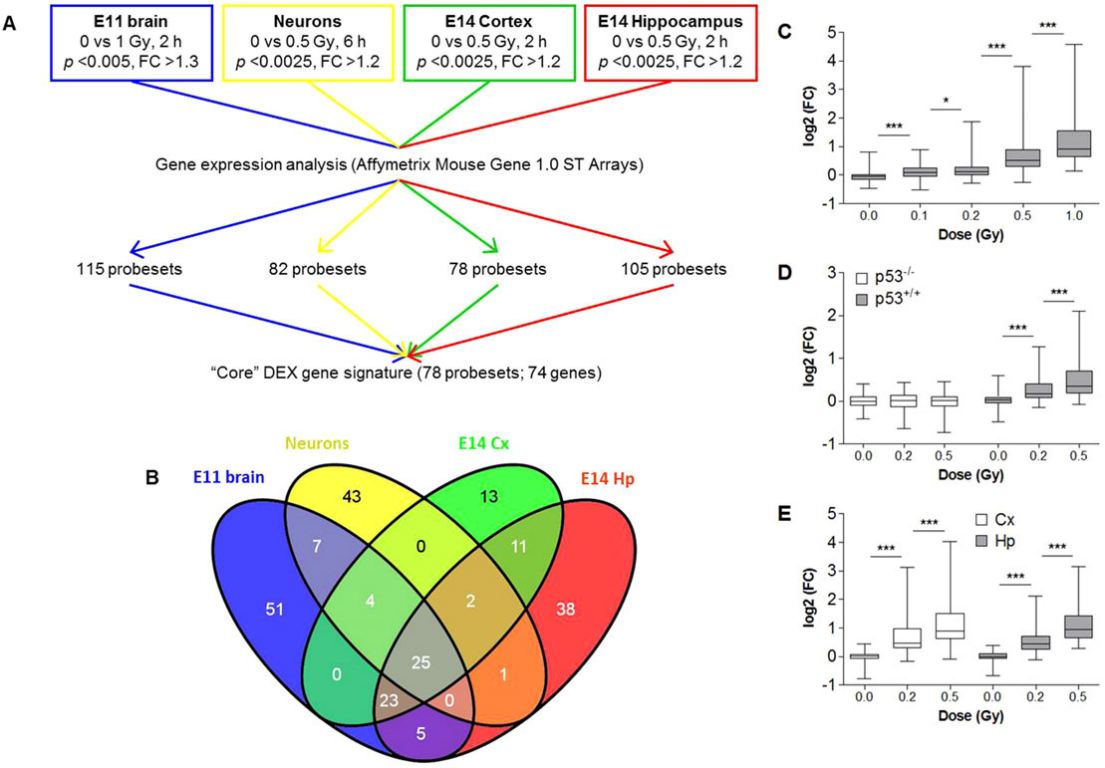
Identification of a core gene signature of radiation-responsive genes in the embryonic mouse brain. (A) Experimental design. (B) Venn diagram showing the overlap of the differentially expressed genes between the different experiments. (C-E) Box plots depicting mRNA expression of core DEX genes in brains from E11 embryos (n = 3) at 2 h post-irradiation (C), primary cortical neuron cultures of 1 DIV (n = 4) at 6 h post-irradiation (D), and cortex and hippocampus of E14 embryos (n = 3) at 2 h post-irradiation (E) with different doses of X-rays. Expression is relative to the expression of sham-irradiated controls. Centerlines show the median, boxes represent the range between first and third quartiles and whiskers represent the highest and lowest values. *p<0.05, ***p<0.0001 (Wilcoxon signed rank test).

CEL intensity files were generated using GeneChip Operating Software and quality tested using the Affymetrix Expression Console. CEL-files were next uploaded to the Partek Genomics Suite (version 6.6) and exon-level data normalization was performed using a customized Robust Multi-array Average algorithm (background correction for entire probe sequence, quantile normalization, log2 transformation of intensity signals). Summarization of exon-level to gene-level data was performed using a one-step Tukey's biweight summarization method as recommended by Affymetrix. Analysis of differentially expressed (DEX) genes was performed using different ANOVA models depending on the experiment. Thresholds for statistical significance for each separate experiment are indicated in Fig. 1A.

### Analysis of alternative exon usage

Detection of alternatively spliced/transcribed (DAS) genes was performed using two parallel approaches. Firstly, we used Alternative Splice ANOVA models in Partek Genomics Suite for all different experiments. To minimize the number of false positives, a filter was used to select for probesets with a log2 expression signal >5.5, which was calculated from the expression level frequency histograms. Probesets with a lower expression signal, but with a *p*-value<0.05, were retained for the analysis. In contrast to what is suggested by Affymetrix, we did retain DEX genes in the Alternative Splice analysis because we were not only interested in alternative splicing events, but also in alternative transcription, as this was shown to occur more frequently than alternative splicing during mouse cerebellar development (Pal et al., 2011). The second method we used was the AltAnalyze software package (Emig et al., 2010), which applies both the Splicing Index and Microarray Detection of Alternative Splicing. Settings for the analysis were as default (e.g. detection above background *p*-value>0.05, probability statistic: moderated *t*-test), except that we used a raw expression threshold >45 (∼5.5 log2 value) and a gene expression cut-off >100 to ensure DEX genes to be taken into account. Results from both methods were then pooled and exon expression plots of genes that were detected as DAS in at least two separate experiments were visually inspected to identify false positive results. Genes of which the splicing pattern was not dose-dependent or which suggested probeset cross hybridization or non-expressed probesets, were therefore omitted (Affymetrix, Technical Note). Genes which were detected as DEX in one experiment and DAS in another, were finally also included in the list of RR genes.

### Reverse transcription and quantitative PCR

Complementary DNA was prepared from total RNA using the GoScript^TM^ Reverse Transcriptase kit (Promega, Leiden, The Netherlands) using 1 µl of random hexamer primers and 3.75 mM MgCl_2_ in 20 µl reactions. Primers used for quantitative PCR are listed in supplementary material Table S1. We used the MESA Green kit (Eurogentec, Seraing, Belgium) according to the manufacturer's instructions using an Applied Biosystems 7500 Fast real-time PCR instrument (Thermo Fisher Scientific). Reaction efficiencies were used for relative quantification using the method as described by Pfaffl (Pfaffl, 2001). *Gapdh* was used as an internal reference gene. For all qRT-PCR experiments the specificity of the primers was validated using a melting curve.

### Gene ontology enrichment analysis

For Gene Ontology (GO) enrichment analysis we used the GOrilla tool (Eden et al., 2009) with the following settings: Organism: *Mus musculus*; Running mode: Two unranked lists of genes (target list: DEX genes; background list: genes expressed above background in at least 30% of all samples); *p*-value threshold: 0.001. The results of this analysis were subsequently reduced using REVIGO (Supek et al., 2011) with default settings. REVIGO serves to remove redundant GO terms. The version of the Gene Ontology used was: go_201304-termdb.obo-xml.gz (which can be found at http://archive.geneontology.org/full/2013-04-01/).

### Transcription factor binding site enrichment analysis

ChIP enrichment analysis (Lachmann et al., 2010) was performed to identify potential transcriptional regulators of RR genes.

### Western blotting

Western blot analysis was performed on total proteins extracted from brains of mice irradiated at E11 and dissected 2 h after irradiation. Proteins were harvested by lysing brain tissues with 200 µl RIPA buffer (50 mM Tris/HCl (pH 8.0), 150 mM NaCl, 1 mM EDTA, 1% Triton X-100, 0.1% SDS) containing protease inhibitor and phosphatase inhibitors cocktail tablets (Roche, Brussels, Belgium). Western blotting was performed using standard procedures with the following primary antibodies: p53-Ser15-P (catalog number 9284, Cell Signaling Technology, Leiden, The Netherlands), total p53 (Pab 240, Abcam, Cambridge, UK), and Gapdh (ab8245, Abcam, Cambridge, UK) as a loading control. For visualization we used chemiluminescence (Clarity Western ECL Substrate, Bio-Rad, Temse, Belgium).

### Identification of p53 binding sites for ChIP

Potential p53 binding sites were identified to be used as target sequences for ChIP-PCR. Because p53 is known to bind both in the promoter as well as in the body of genes (Li et al., 2012a; Riley et al., 2008) we downloaded genomic sequences starting from 5000 base pairs before the transcription start site until the end of the transcript. These sequences were then used for a matrix scan analysis (http://rsat.ulb.ac.be/rsat/) using the MA0106.1 (TP53) matrix from the JASPAR database. Parameters used were: *Mus musculus*-specific background model estimation (Markov order = 0) and scanning options were used as default with a *p*-value threshold set at 10^−4^. Primers for PCR (supplementary material Table S2) were designed to span the predicted binding sites (supplementary material Fig. S3).

### Chromatin immunoprecipitation

ChIP was performed on brains from E11 mice that were pooled per litter. For each condition, three independent litters were used for ChIP with the SimpleChIP Enzymatic Chromatin IP kit (Magnetic beads) (Cell Signaling Technology, Danvers, MA, USA) according to the manufacturer's protocol with minor modifications.

Brains were dissected at 2 h after (sham-)irradiation and fixed in 1% formaldehyde (252549, Sigma-Aldrich, Bornem, Belgium) supplemented with Protease Inhibitor Cocktail (P8340, Sigma-Aldrich, Bornem, Belgium) for 10 min at room temperature on an orbital shaker (300 r.p.m.). Formaldehyde was quenched using glycine, tissues were rinsed twice with phosphate buffered saline (PBS) and collected in PBS containing phenylmethanesulfonyl fluoride (93482, Sigma-Aldrich, Bornem, Belgium). DNA was digested using 20 µl of micrococcal nuclease (20 gel units/µl) for 20 min at 37°C. Chromatin was isolated and DNA-protein complexes were immunoprecipitated using antibodies against phospho-p53 (Ser15) (catalog number 9284, Cell Signaling Technology, Leiden, The Netherlands) or mouse IgG provided with the kit. Purified DNA fragments were amplified by PCR (Taq & Load Mastermix, MP Biomedicals, Santa Anna, CA, USA) using 34 cycles. PCR products were run on a 1.5% agarose gel and the intensity of the bands was quantified using ImageJ. For normalization, densitometric signals from IgG were subtracted from p53-Ser15-P signals for each sample.

### Gene set enrichment analysis (GSEA)

GSEA was performed using default settings. Because of the small amount of samples (n = 2–3), we used 1000 permutations (gene_set), with a weighted enrichment statistic and a signal-to-noise metric for gene ranking. As gene sets, we used all significant differentially expressed genes (ANOVA *p*<0.001 and FC>|2|) at any of the used developmental stages (E9, E10, E11, E14, E16), as well as gene sets that were downloaded from the MSigDB database (Cell Cycle, Apoptosis, DNA Repair, Brain Development, Neuron Differentiation). For the radiation-responsive gene set, only DEX genes were used since GSEA does not take alternative splicing into account.

## RESULTS

### Identification of a radiation-responsive gene signature in the developing brain

The initial objective of this study was to perform a meta-analysis in order to identify a bona fide set of genes which mediate the early effects of exposure to ionizing radiation in the developing brain. To this end, we combined whole genome expression data from different experiments that had previously been performed in our lab as explained in Fig. 1A. For each of the individual analyses, we identified DEX probesets which resulted in the identification of 115, 82, 78, and 105 DEX probesets in the E11 brain, primary cortical neuron cultures and the E14 cortex and hippocampus, respectively (Fig. 1A). We next considered only those probesets that were DEX in at least two of these experiments, resulting in a signature consisting of 78 probesets, corresponding to 74 individual genes (Fig. 1B; supplementary material Table S3). Interestingly, all of these genes were upregulated after radiation exposure. In all of the separate experiments, the average expression of DEX genes was dose-dependently induced even at the lowest doses of 0.1 Gy in the whole brain at E11 (Fig. 1C) and of 0.2 Gy in primary cortical neuron cultures (Fig. 1D) as well as in the E14 cortex and hippocampus (Fig. 1E).

### Radiation-induced alternative splicing

**Fig. 2.**
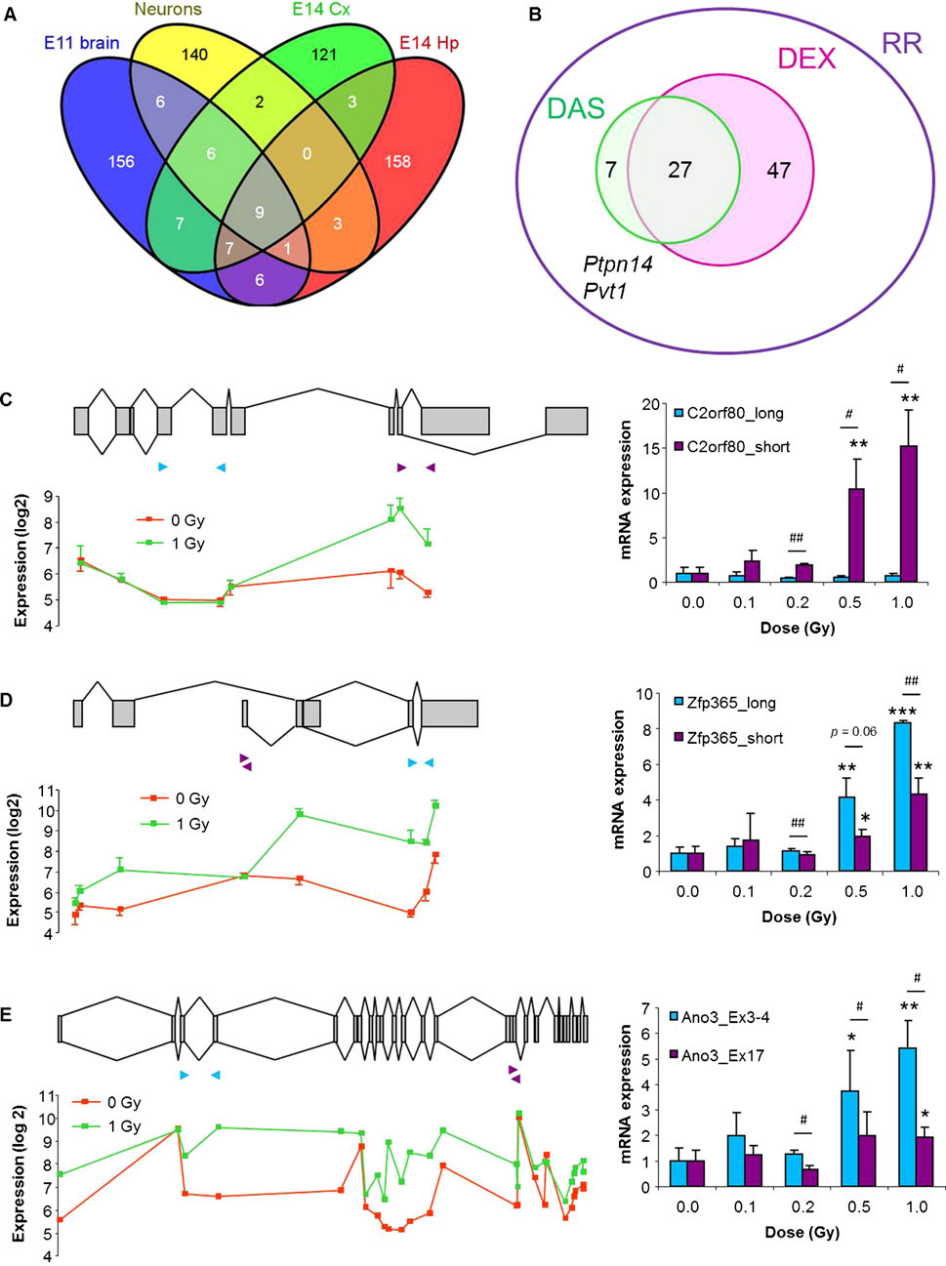
Identification and validation of radiation-induced alternatively spliced genes. (A) Venn diagram showing the overlap of the DAS genes between the different experiments. (B) Venn diagram showing the overlap between radiation-induced DEX and DAS gene signatures. The combination of DEX and DAS genes, together with *Ptpn14* and *Pvt1*, which were DEX in one experiment and DAS in another, is then the radiation-responsive (RR) gene signature. (C–E) Validation of radiation-induced alternative splicing of *D630023F18Rik*/*C2orf80* (C), *Zfp365* (D), and *Ano3*/*Tmem16c* (E). Left panels show exon organization of the gene, with exons shown as grey boxes and known variants indicated by connecting lines (top), and log2 microarray expression signals for each separate probeset (below). Right panels show mRNA expression of different transcript variants as assessed by qRT-PCR from brains of in utero (E11) irradiated mice (n = 3). Transcript-specific primers are indicated with arrowheads on the left panels. Asterisks indicate significant difference compared to 0 Gy (*p<0.05; **p<0.01; ***p<0.001). For comparisons between transcript variants a paired Student's *t*-test was used (^#^*p*<0.05; ^##^*p*<0.01). Error bars represent s.e.m.

Alternative splicing is a very frequent event during embryonic development (Revil et al., 2010) and especially in the brain (Grosso et al., 2008; Yeo et al., 2004; Zheng and Black, 2013), where it is important for neurogenesis, neuronal migration, synaptogenesis, and neuronal differentiation (Norris and Calarco, 2012). Moreover, recent studies have shown that exposure to ionizing radiation can affect alternative splicing of a number of genes (Muñoz et al., 2009; Sprung et al., 2011). Therefore, we also analyzed radiation-induced DAS genes from these experiments and identified 50 genes that showed transcript variation after X-irradiation in at least two separate experiments (Fig. 2A). After additional visual inspection of their exon intensity signals in a genomic context, we omitted 16 false positives because of probeset cross hybridization, non-expressed probesets or lack of dose-dependence. Thus, 34 genes were finally retained as being DAS. Most of these were also DEX at the gene level, although we identified another seven genes which were detected as being only DAS, but not DEX, in at least two of our experimental settings (Fig. 2B). The latter thus represent genes of which the expression levels of only one or a few exons were changed after irradiation, and would have been missed if we had only analyzed the data at the gene level. Furthermore, two genes (*Ptpn14* and *Pvt1*) were identified as DEX in one experiment and DAS in another. The union of DEX genes (74 genes), DAS genes (7 genes) and *Ptpn14* and *Pvt1* were finally considered as the radiation-responsive (RR) gene signature (83 genes, Fig. 2B; supplementary material Table S3).

To confirm the microarray results regarding alternative splicing, we used exon- and variant-specific qRT-PCR to validate a few candidate genes in brains from E11 mice that were irradiated with different doses of X-rays. *D630023F18Rik* (further referred to as *C2orf80*) has several known transcript isoforms depending on the usage of alternative 3′ splice sites or an alternative 3′ exon (Fig. 2C). Our microarray data suggested that exposure to radiation results in the specific induction of a not yet identified short isoform. This was confirmed by qRT-PCR using primers specific for the 5′ part (long) or the 3′ part (short) of the gene, showing that there was a dose-dependent increase in the expression of the 3′ part only (Fig. 2C). Another DAS gene was *Zfp365*, of which different isoforms exist, depending on the usage of an alternative promoter or a bleeding exon (Fig. 2D). According to the microarrays, both long and short isoforms are expressed in the embryonic brain, but radiation exposure specifically induced the long isoform (Fig. 2D). qRT-PCR experiments using transcript-specific primers partially confirmed these results. Indeed, we found a dose-dependent induction of both variants, although the induction of the short variant was significantly reduced compared to the long variant (Fig. 2D). Finally, *Ano3* (also known as *Tmem16c*) has two known isoforms, one of which is a truncated transcript that lacks the eight most distal exons (Fig. 2E). Of this gene, several exons showed a deviating expression profile after radiation, indicating the induction of unknown splice variants (Fig. 2E, left panel). This was again confirmed by qRT-PCR, showing a dose-dependent increase in the expression of isoforms containing exons 3 and 4, whereas isoforms containing exon 17 were significantly less induced after radiation exposure (Fig. 2E).

### Early radiation-responsive genes are mostly p53 targets enriched in canonical p53-regulated pathways

**Fig. 3.**
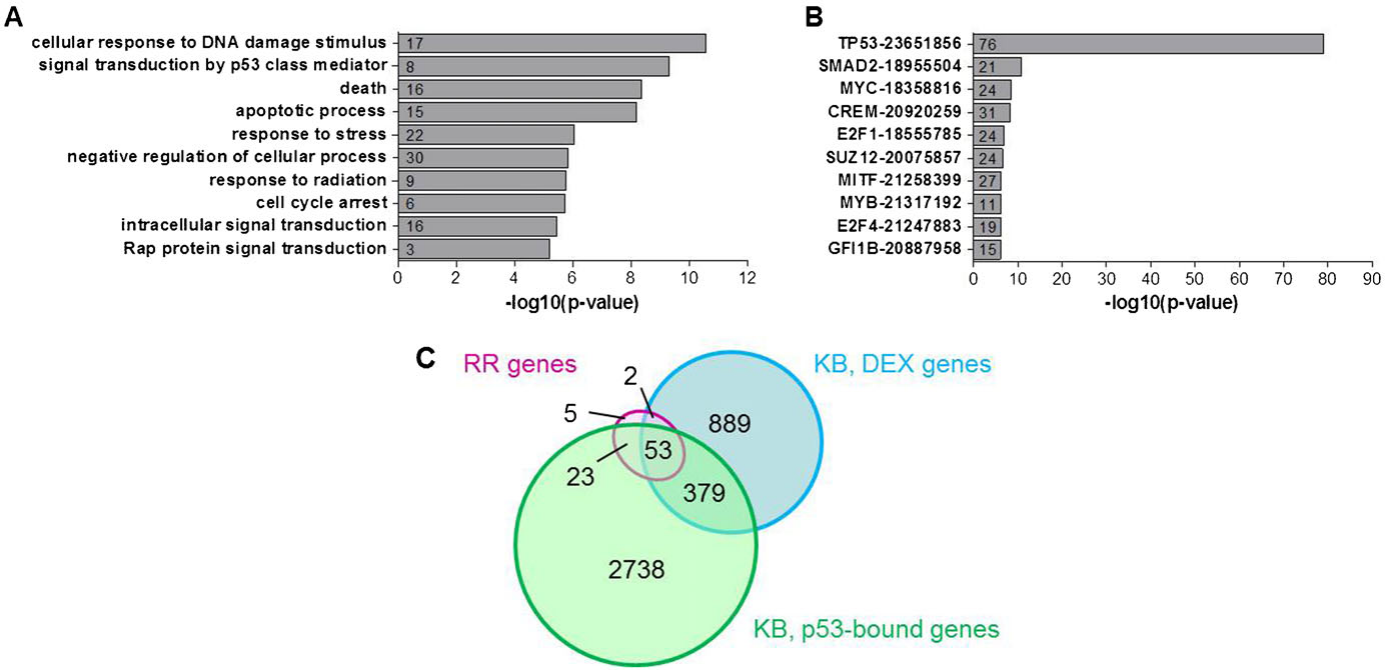
Radiation-responsive genes are involved in classical p53-mediated DNA damage response pathways. (A) GO enrichment analysis of RR genes. (B) ChIP enrichment analysis of RR genes. Numbers in bars indicate the number of genes from the RR gene signature that are represented in the respective gene lists. Numbers behind transcription factors indicate PubMed IDs of the respective studies. (C) Overlap between our RR gene signature, and DEX and p53-bound genes in doxorubicin-treated MEFs (Kenzelmann Broz et al., 2013).

Gene ontology enrichment analysis showed that RR genes are enriched in classical radiation response pathways such as apoptosis, DNA damage response and cell cycle arrest (Fig. 3A) which are well known to be regulated by p53. In order to verify this, we analyzed the overrepresentation of the gene signature in gene lists from genome-wide ChIP experiments using Chip Enrichment Analysis (ChEA) (Lachmann et al., 2010). This showed that p53 was by far the most significant transcription factor predicted to be involved in the regulation of these genes (Fig. 3B) as we already observed in previous experiments on E13 mouse brains (Verheyde et al., 2006). Other predicted regulators of RR genes were, among others, Smad2, which localizes to DNA double strand breaks and cooperates with p53 in the DNA damage response (Wang et al., 2013), as well as c-Myc and E2f1, which have been shown to stabilize p53 via mechanisms similar to those in response to DNA damage (Lindström and Wiman, 2003). Yet, the complete lack of radiation-induced expression of DEX genes in p53^−/−^ cortical neuron cultures (Fig. 1D), suggests that if other factors were involved in transcriptional regulation of these genes, they would still depend on p53 as a co-factor. The ChEA analysis further showed that there was a very large overlap (Fig. 3B,C) between our RR signature and genes that were bound by p53 and/or DEX after treatment of mouse embryonic fibroblasts (MEFs) with the DNA damaging agent doxorubicin (Kenzelmann Broz et al., 2013). It is interesting to note that while only a small fraction (33%) of DEX genes in doxorubicin-treated MEFs are bound by p53, almost all (92%) of the genes from our signature were p53-bound in these cells, indicating that they are genuine transcriptional targets of p53.

### Validation of novel p53 target genes

**Fig. 4.**
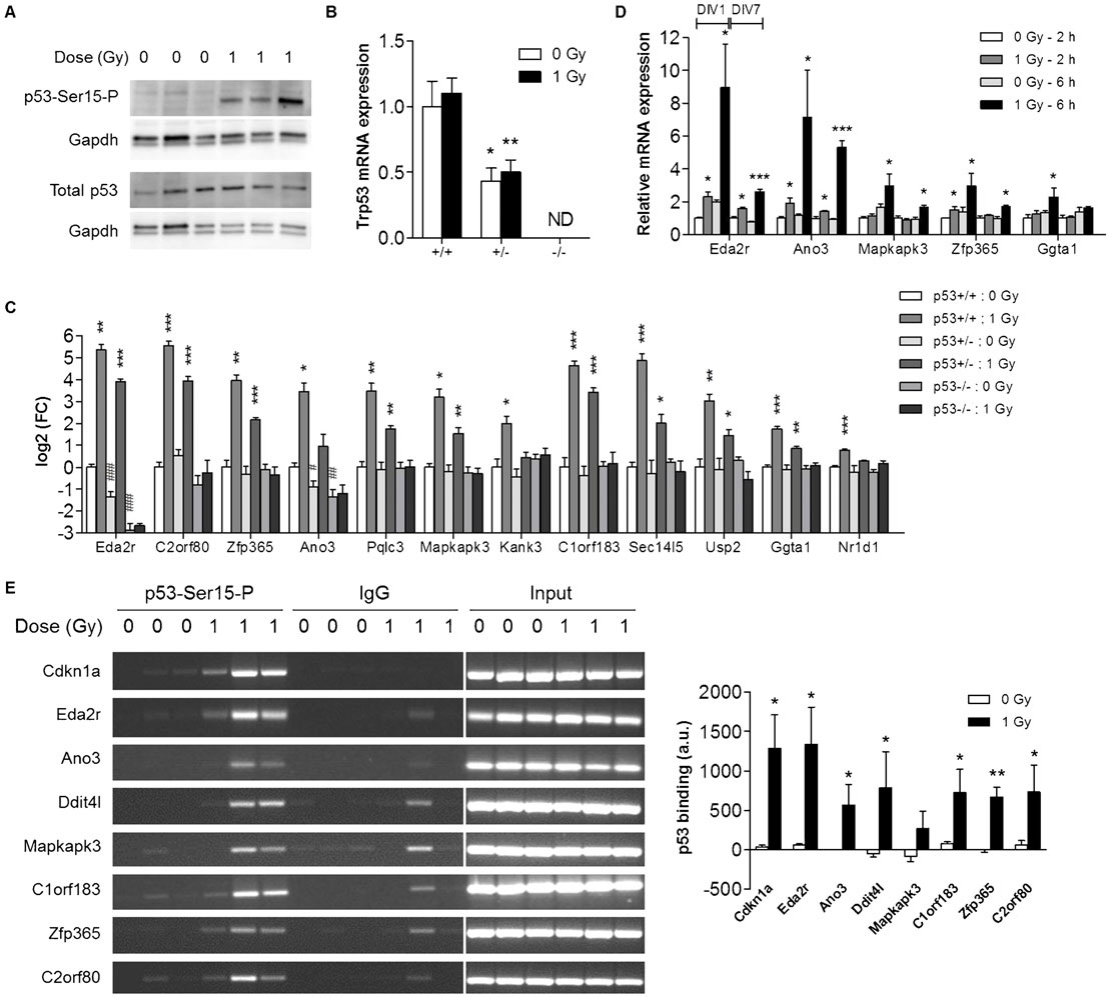
p53-dependent expression of novel target genes. (A) Western blotting was performed using antibodies against the phosphorylated (p53-Ser15-P) as well as the total form of p53. (B,C) mRNA expression was determined by qRT-PCR in E11 brains from p53^+/+^, p53^+/−^ and p53^−/−^ littermates (n = 4) at 2 h post-irradiation. Please note the logarithmic scale of the Y-axis in B. *p<0.05; **p<0.01; ***p<0.001 for the difference between control and irradiated mice from the same genotype (Student's *t*-test). ^#^*p*<0.05; ^##^*p*<0.01; ^###^*p*<0.001 for difference with control p53^+/+^ mice (Student's *t*-test). (D) mRNA expression was determined by qRT-PCR in primary cortical neuron cultures (1 DIV and 7 DIV) at 2 h and 6 h post-irradiation (n = 3–4). *p<0.05; ***p<0.001 (Student's *t*-test). (E) ChIP-PCR was performed on pooled brains from individual litters (n = 3) of in utero irradiated mice at 2 h post-irradiation. Densitometric analysis of gel electrophoresis bands was performed using ImageJ. *p<0.05; **p<0.01 (paired Student's *t*-test). In all panels data indicate mean+s.e.m. ND, not detected; DIV, days *in vitro*.

For validation experiments, we selected random genes based on their novelty as potential p53 targets. We also further analyzed the known p53 target *Eda2r*, because it was the most significantly regulated gene in each of the four separate experiments, indicating the importance of this gene in the p53-mediated response in the brain. The early activation of p53 at 2 h post-irradiation was first demonstrated by western blotting using an antibody against the phosphorylated form of p53 at the serine 15 residue (Fig. 4A), which is the main target of the DNA damage response to radiation (Meek, 2009). Next, we performed qRT-PCR on brains from control and 1 Gy X-irradiated p53^+/+^, p53^+/−^ and p53^−/−^ littermates at E11. *Trp53* gene expression levels in p53^+/−^ mice were about half of those in wild-type mice, whereas in p53^−/−^ mice it was not at all detectable (Fig. 4B). For all of the tested target genes, we found a significant upregulation, ranging from 1.7-fold (*Nr1d1*, *p* = 0.0002) to 46-fold (*C2orf80*, *p* = 0.0008), in the irradiated brains from p53^+/+^ mice, which was attenuated in p53^+/−^ mice and completely abrogated in p53^−/−^ mice (Fig. 4C). This shows that p53 gene dosage, and therefore the amount of available p53, is important for the extent of the transcriptional regulation of these genes. These data further revealed that *Eda2r* and *Ano3* expression decreased with decreasing p53 gene dosage in non-irradiated control animals (Fig. 4C), suggesting that they are constitutively regulated by p53. In contrast, the other tested genes seem to become activated by p53 only in response to stress signals such as DNA damage, at this early time point in brain development.

We also validated the expression levels of several genes after irradiation of immature and maturing primary cortical neuron cultures. These experiments again confirmed our microarray data by showing an upregulated expression of radiation-responsive gene isoforms in the irradiated cultures (Fig. 4D; supplementary material Fig. S1), which could be prevented by prior treatment of the cells with the p53 inhibitor α-pifithrin (supplementary material Fig. S1). Interestingly, we also observed that the transcriptional effect of radiation exposure was less pronounced in the more mature cultures compared to immature cultures (Fig. 4D), in line with a previous study which demonstrated that DNA damage-dependent p53 activation is more robust in immature compared to mature cortical neurons (Martin et al., 2009). To obtain better insight in the cell type specificity of this p53-mediated transcriptional response, we also evaluated radiation-induced gene expression in astroglial cell cultures. This showed that the p53 response was not induced in these cells (supplementary material Fig. S2), suggesting that it is neuron-specific.

Finally, we performed ChIP-PCR on a selection of genes to assess radiation-induced binding of p53 to their promoters. We found that for all of the tested genes p53 binding was very low in non-irradiated brains and was substantially increased 2 h after irradiation (Fig. 4E). Interestingly, in the cases of *C2orf80* and *Zfp365*, our data indicate that the alternative transcription we observed after irradiation (Fig. 3A,B) resulted from the binding of p53 to an alternative promoter of the gene (supplementary material Fig. S3), as could also be predicted from our microarray results.

### Radiation-responsive genes are significantly enriched during brain development

Several genome-wide studies of p53 target gene expression in different non-neuronal cell types have demonstrated that p53 targets are enriched in genes involved in functions such as neuron differentiation, nervous system development and axon guidance (Botcheva et al., 2011; Kenzelmann Broz et al., 2013; Kracikova et al., 2013; Smeenk et al., 2008; Yoon et al., 2002). Moreover, as previously mentioned, p53 is believed to be important for neuron differentiation and brain development. Therefore, we analyzed gene expression changes in control mice with the purpose of evaluating specifically the expression of RR genes at five different stages of embryonic brain development (E9, E10, E11, E14, E16).

**Fig. 5.**
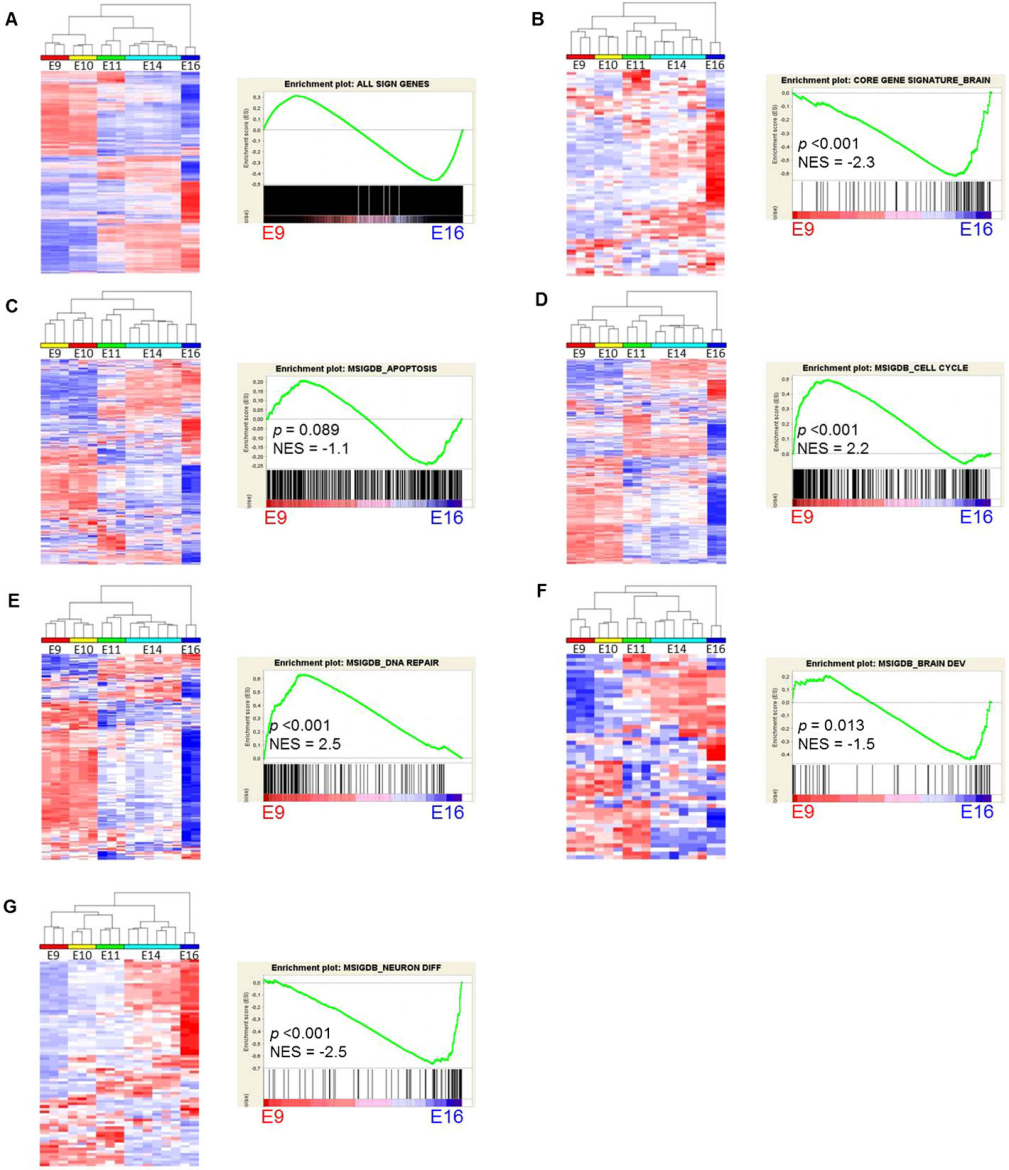
Gene expression profiles of DEX genes and gene sets of different functional pathways in the embryonic brain. (A–G) Unsupervised hierarchical clustering (left panels) and GSEA analysis (right panels) of all significant genes (A), DEX gene signature (B), apoptotic genes (C), cell cycle genes (D), DNA repair genes (E), brain development genes (F) and neuron differentiation genes (G). For the different functional classes, we used gene lists from the MSigDB database. NES, normalized enrichment score.

In this dataset, no less than 40% of all expressed genes had different expression levels (ANOVA *p*<0.001 and FC >|2|) at some point between E9 and E16 (Fig. 5A), demonstrating that a very large fraction of the transcriptome is modulated during the earliest stages of brain development. The principal component analysis plot (supplementary material Fig. S4A) further shows that gene expression changes are very dynamic and occur over short time periods, especially between E10 and E11 and between E14 and E16. This corresponds to the time points when neurogenesis is initiated in most of the brain regions (E11), which peaks at E14, before declining again in the telencephalon, diencephalon and midbrain (Götz and Huttner, 2005). Interestingly, compared to the total number of expressed genes, the fraction of radiation-induced DEX genes that were differentially expressed during early brain development was significantly larger [63%, *p* = 7.6×10^−5^ (Chi square, Yates' corrected)]. Moreover, when we also included genes that were considered as alternatively spliced during development, no less than 92% (68 out of 74) of DEX genes changed in expression at some point between E9 and E16. Therefore, p53 seems to preferentially target developmentally regulated genes after radiation exposure of the developing brain.

Since many of the genes in our signature were only poorly characterized, and in view of their aforementioned developmental regulation, we hypothesized that these genes could be functionally important for normal brain development. We therefore analyzed gene expression profiles of different functional classes of genes using GSEA (Subramanian et al., 2005), under the assumption that similarly expressed genes are likely to have similar biological functions, a concept known as “guilt by association”. We first performed GSEA analysis on all developmentally regulated genes, which showed that these are almost equally enriched in either E9 or E16 brains (Fig. 5A). In contrast, radiation-induced DEX genes were significantly enriched in E16 brains (Fig. 5B). However, similar to the totality of significant genes, genes involved in apoptosis were equally enriched at E9 and E16 (Fig. 5C), whereas cell cycle and DNA repair-related genes were significantly enriched in E9 brains (Fig. 5D,E). Interestingly, most of the genes from our signature that are developmentally downregulated (left part of the GSEA-plot, Fig. 5B), belong to pathways related to apoptosis (*Lrdd*, *C11orf82*/*Noxin*, *Apaf1*, *B230120H23Rik*, *Tnfrsf10b*), cell cycle regulation (*Gtse1*, *Ckap2*) and DNA repair (*Polk*, *Ercc5*, *Rnf169*). On the other hand, genes involved in brain development (Fig. 5F) and especially neuron differentiation (Fig. 5G) showed a significant enrichment in E16 brains, comparable to the DEX gene signature. These results are consistent with two recent studies in mice which showed that mitosis, cell cycle and DNA repair pathways were enriched in embryonic brains compared to post-natal and adult brains, whereas genes involved in synaptic transmission and ion homeostasis increased in expression during development (Dillman et al., 2013; Pramparo et al., 2011). Another recent study investigated genome-wide spatiotemporal transcriptional profiles of the mid-gestational human brain (Miller et al., 2014). Importantly, we found a very good correspondence between our data and those of the human embryonic brain. Miller et al. identified 42 modules of co-expressed genes, two of which were especially consistent between different data sets. Supplementary material Fig. S5 shows the genes from those two modules, one of which is enriched in germinal layers and decreases in expression with age (yellow, C38). This module is enriched in functions related to mitosis and spindle formation (Miller et al., 2014) and contains *Ckap2*, *Gtse1* and *C11orf82*/*Noxin* from our gene signature (supplementary material Fig. S5A). Another gene module (brown, C22) is enriched in post-mitotic neurons from the cortical plate, and increases with age. From our signature, *Ampd2*, *Bbc3*, *Cgref1*, *Cpt1c* and *Baiap2* all belong to this module (supplementary material Fig. S5B). Notably, as in humans, the genes from the yellow module are also enriched in the early embryonic mouse brain whereas genes from the brown module are enriched in the late embryonic mouse brain (supplementary material Fig. S5C). Together, these data suggest that radiation-induced p53 activation in the embryonic brain induces a transcriptional profile which is reminiscent of differentiating neurons and that the normal, physiological function of the genes in our signature is more related to brain development or neuron differentiation, rather than the DNA damage response.

**Fig. 6.**
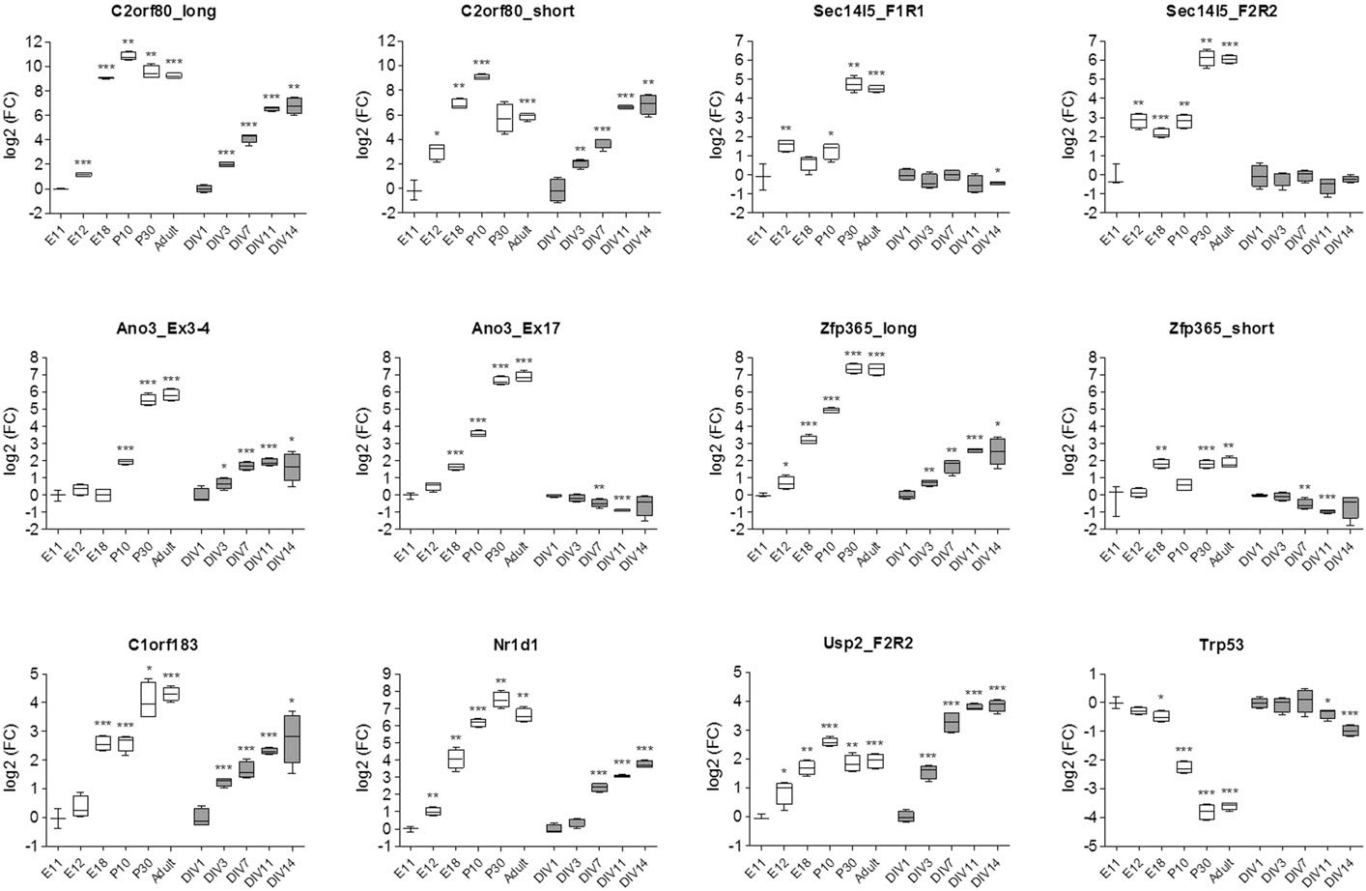
mRNA expression of selected RR genes and *Trp53* during mouse brain development and in differentiating primary cortical neuron cultures. mRNA expression was determined by qRT-PCR in brains from mice at embryonic days 11, 12 and 18, postnatal days 10 and 30, and at adult age (white boxes) and in primary cortical neuron cultures at days 1, 3, 7, 11 and 14 of culture (gray boxes). For all conditions n = 4 except for E11 where n = 3. Data are expressed as fold changes (log2 scale) relative to E11 (for *in vivo* data) or DIV1 (for *in vitro* data). For explanation of the box and whisker plots, see Fig. 1E, embryonic day; P, post-natal day; DIV, days *in vitro*; FC, fold-change. *p<0.05; **p<0.01; ***p<0.001 (Student's t-test).

To further validate these observations, we performed qRT-PCR on a selection of genes from mouse brains at an expanded set of pre- and post-natal developmental stages, as well as in cultures of primary cortical neurons at different days *in vitro* (DIV). These experiments showed that all of the tested genes were indeed induced during brain development and *in vitro* neuronal differentiation (Fig. 6). Overall, the expression levels of these genes gradually increased during development, before reaching maximal values at either post-natal day 10 (*C2orf80* and *Usp2*) or 30 (*Sec14l5*, *Ano3*, *Zfp365*, *C1orf183* and *Nr1d1*). Furthermore, we observed that the developmental expression pattern of different transcript variants of these genes was not always comparable. Notably, the radiation unresponsive transcript variants of *Zfp365*, *Ano3* (Fig. 6) and *Usp2* (not shown) showed deviating expression levels with either no change, or decreased expression during development or differentiation. Thus, all of the tested p53-responsive variants increased in expression during development and differentiation in contrast to p53 itself, which decreased over time (Fig. 6). This indicates that these genes are not regulated by p53 under normal physiological conditions but only in response to a cellular stressor such as ionizing radiation.

### Regulation of radiation-responsive genes in neurological disorders

**Fig. 7.**
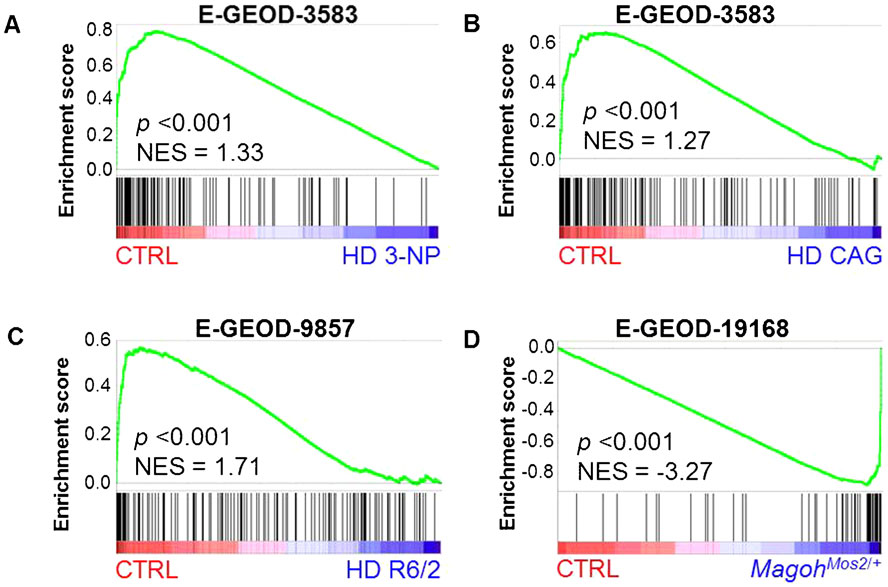
Radiation-responsive DEX genes are highly regulated in mouse models of Huntington disease and microcephaly. GSEA shows significant downregulation of DEX genes in three different mouse models of Huntington disease (A–C) and significant upregulation of DEX genes in a mouse model of microcephaly (D). Gene Expression Omnibus accession numbers for each data set are shown above the graphs.

Several neurodegenerative diseases and neurological disorders have been associated with DNA damage or disturbances in the DNA damage response in neuronal cells. For instance, HD is a neurodegenerative disease caused by an expansion of CAG triplicate repeats in the Huntingtin gene (*Htt*). HD pathogenesis is very complex, with many cellular pathways being affected, but one of its hallmarks is the occurrence of DNA damage, resulting in the activation of p53 and the induction of DNA damage response proteins prior to the accumulation of Htt protein aggregates (Illuzzi et al., 2009). Therefore, we analyzed the expression profile of the DEX gene signature in different mouse models of HD by GSEA. To our surprise, we found that expression of these genes was significantly downregulated in all three HD models compared to control mice (Fig. 7A–C). Again, this suggests that our gene signature is primarily involved in neuronal functions since it was shown that mutant Htt downregulates expression of neuronal genes by increasing the nuclear translocation of the transcriptional repressor Rest/Nrsf (Zuccato et al., 2003).

Another neurological disorder which is very often associated with DNA repair deficiency is microcephaly (McKinnon, 2009). In humans microcephaly is defined as a head circumference of more than two standard deviations below the average for age and gender. Many genetic and environmental factors can cause microcephaly, one of which is prenatal radiation exposure, especially during weeks 8 to 15 of pregnancy (Otake and Schull, 1993; Otake and Schull, 1998). In animal models, microcephaly is defined as a reduced brain size and it has been recapitulated in a number of genetic mouse models as well as by prenatal exposure to ionizing radiation (Kitamura et al., 2001; Sun et al., 1995). In fact, we also found that radiation exposure of pregnant mice at E11 to doses of 0.66 Gy and higher, led to a dose-dependent decrease in brain and body size of the pups (Verreet et al., unpublished results). One of the genetic microcephaly models is the *Magoh^Mos2^*^/+^ mouse (Silver et al., 2010) which is characterized by a reduced body size and microcephaly. Interestingly, these mice display increased neuronal DNA damage, premature neuronal differentiation and apoptosis at early stages of brain development, possibly as a result of mitotic defects. Despite the rather severe phenotype, gene expression changes in these mice were quite modest, but, as is shown by GSEA (Fig. 7D), correlated extremely well with gene expression changes in the irradiated embryonic brain. Altogether, the extensive regulation of our gene signature at different developmental stages and in differentiating cortical neurons, as well as in several neurological mouse models further supports their importance for the proper functioning and development of the mouse brain.

## DISCUSSION

In this study, we combined genome-wide gene expression data from different experiments to identify a gene signature of bona fide radiation-responsive genes in the developing brain. This signature was found to be enriched in genes which belong to classical pathways of the DNA damage response and predicted to be mainly regulated by p53. We also identified several genes expressing different transcript variants after exposure to radiation. Moreover, we could show that radiation-induced transcript variation depends on p53 activity, since treatment of cortical neuron cultures with the p53 inhibitor α-PFT prior to radiation exposure prevented the upregulation of radiation-responsive transcript variants (supplementary material Fig. S1), and binding of p53 to alternative promoters of *C2orf80* and *Zfp365* was significantly enhanced after irradiation (Fig. 4E). As reviewed by Riley et al., four sets of experimental criteria are commonly used for identification of p53-regulated genes: (1) presence of a p53 responsive element (RE) in or near the gene; (2) up- or downregulation of the gene by wild-type, but not mutant p53; (3) transcriptional regulation of a test gene (e.g. luciferase) by a cloned RE, and (4) validation of p53 binding to the RE using ChIP or gel shift assays (Riley et al., 2008). Yet, based on our and previous results (Nicholls et al., 2004; Sprung et al., 2011), we recommend to broaden the second criterion to consider also p53-dependent alternatively spliced or transcribed genes as genuine p53 targets.

One of the important findings of our study is the very high overlap between p53-bound and –regulated genes (Fig. 3C). Although we only validated p53 binding for a few novel targets, the very high overlap of our signature with other genome-wide screens of p53 binding after full p53 activation suggests that most, if not all, of them are bona fide p53 targets. In all of those studies large numbers of genes were transcriptionally regulated and/or bound by p53 (Kenzelmann Broz et al., 2013; Menendez et al., 2009; Nikulenkov et al., 2012; Smeenk et al., 2008), with only very limited overlap between regulated and bound genes indicating that also other factors were involved in directly regulating gene expression. The moderate doses of radiation we used in our experiments were therefore likely to generate a more specific p53-mediated response, which does not (directly) involve other transcription factors such as the other p53 family members, p63 and p73, as was the case in some of the aforementioned studies (Kenzelmann Broz et al., 2013; Smeenk et al., 2008). Indeed, this is also shown by the lack of a transcriptional response in p53^−/−^ brains and cells, or after treatment with the p53 inhibitor α-PFT.

Although we found a significant enrichment of functions related to the DNA damage response, this enrichment is based on less than half of the genes in our signature. The other genes either do not belong to significantly enriched functional pathways or have not yet been functionally characterized. Our observation that, unlike genes involved in the classical p53-regulated pathways, most of our signature genes are significantly upregulated during (early) brain development and neuronal differentiation, suggests that their primary role is related to cellular differentiation or brain-specific functions. This is in correspondence with results from genome-wide p53 binding screens in different non-neuronal cell types, which showed that p53-bound genes are enriched in functions such as general differentiation and development, neuron differentiation and axon guidance (Akdemir et al., 2014; Botcheva et al., 2011; Kenzelmann Broz et al., 2013; Kracikova et al., 2013; Menendez et al., 2009; Nikulenkov et al., 2012; Smeenk et al., 2008; Yoon et al., 2002). Interestingly, two recent publications showed that DNA damage-mediated p53 activation in mouse embryonic stem cells (mESCs) preferentially induces genes which are associated with differentiation and developmental processes rather than in cell cycle or apoptosis (Lee et al., 2012; Li et al., 2012a). Thus, we hypothesize that radiation-induced activation of differentiation-related genes, results in premature differentiation of cells in the embryonic brain. DNA damage-induced differentiation is a well-known defense mechanism to prevent (stem) cells with excessive damage from obtaining a malignant phenotype (Lee et al., 2010; Wang et al., 2012). However, as mentioned in the introduction, the effects of p53 on cellular differentiation are cell type-dependent, with indications that p53 inhibits differentiation in neural stem cells whereas it promotes neural gene expression and neurite outgrowth in post-mitotic cells. Therefore, it will be important to further investigate the exact regulation of p53 and its target genes at the cellular rather than the tissue level. The fact that the p53 transcriptional response was diminished in more mature cortical neuron cultures compared to immature cultures, and was completely absent in astroglial cells, indicates that the transcriptional effects we observed *in vivo* are restricted to certain cell type(s), including differentiating neurons.

The validity of our hypothesis that p53 activation may result in premature neuronal differentiation, is further strengthened by the analysis of the expression of RR genes in mouse models of neurological disorders. Indeed, both the downregulation in HD as well as the upregulation in an embryonic microcephaly model displaying premature neuronal differentiation, indicate that RR genes may have important roles in normal brain function and differentiation. Microcephaly is the only congenital malformation of pre-natal radiation exposure and it is often associated with increased DNA damage, mitotic spindle defects and subsequent premature neuronal differentiation. The very strong overlap between gene expression profiles and phenotypes of our prenatally irradiated mice and the microcephalic *Magoh^Mos2/+^* mice (Silver et al., 2010) suggests that (1) the gene expression changes in *Magoh^Mos2^*^/+^ mice result, at least partly, from p53 activation and (2) that these changes are responsible for the similar phenotypes. In this respect, it is interesting to note that several genes from our signature have been previously shown to be involved in spindle and/or microtubule formation (*Apaf1*, *AW555464/Cep170b*, *Bloc1s2*, *Ckap2*, *Gtse1*, *Usp2*, *Zfp365*). Also, one of the genes which was upregulated in *Magoh^Mos2^*^/+^ mice and irradiated brains is *Cpt1c*, a brain-specific regulator of fatty acid synthesis which is developmentally upregulated after birth (Carrasco et al., 2013). A brain-specific gain-of-function model of this gene displayed post-natal microcephaly and growth retardation (Reamy and Wolfgang, 2011). To better understand the possible function of p53 in the development of microcephaly, it would be interesting to investigate the effects of a double *p53*/*Magoh* deficiency on the spindle formation, neuronal differentiation and the size of the brain.

Several of the genes from our signature have previously been shown to be involved in brain-related functions such as neurite outgrowth (*Baiap2*, *Cdc42bpg*, *Igdcc4*), focal adhesion dynamics (*Arap2*), neuronal differentiation (*Btg2*, *Foxo3*, *Gne*, *Grhl3*), calcium sensing (*Hpcal1*), and synaptic transmission (*Rap2a*). Moreover, *Ampd2* has recently been shown to be mutated in pontocerebellar hypoplasia, a progressive neurodegenerative disorder (Akizu et al., 2013), whereas mutations in *Ano3* were linked with craniocervical dystonia (Charlesworth et al., 2012). We believe that elucidating the function of *Ano3* in brain development may be of importance for a number of reasons. *Ano3* seems to play an important role in the mammalian brain since it was found to be a hub gene in weighted gene co-expression analyses of the caudate nucleus (Oldham et al., 2006), whereas it was also identified as a hub gene in a module of co-expressed genes that had low expression in the embryonic cortex and hippocampus in humans, but progressively increased with age (Kang et al., 2011). Moreover, a number of SNPs were found to be associated with late-onset Alzheimer's disease in a GWAS study (Briones and Dinu, 2012). Finally, a recent study showed that Ano3 is involved in pain processing in the rat by facilitating Na^+^-activated K^+^ currents in sensory neurons (Huang et al., 2013). All of these data indicate that *Ano3* is important in the mammalian brain, which is also suggested from its increasing gene expression pattern at different stages of brain development and brain-specific expression at adult age (data not shown). The fact that radiation exposure results in specific induction of certain *Ano3* transcript variants, which are specifically induced during neuron differentiation, adds to its attractiveness for further study.

Another interesting and currently unknown p53 target is *C2orf80*, which showed a very high upregulation (>50-fold) of a short isoform early after irradiation. Moreover, both short and long isoforms are very strongly induced during *in vivo* brain development (∼2000-fold) and *in vitro* neuronal differentiation (>100-fold). So far, this gene has only been mentioned in a couple of research articles. In one of these, *C2ORF80* along with *STMN3A*, a gene involved in neurite outgrowth, were the most significantly affected after knockdown of a psychosis susceptibility gene in human neural progenitor cells (Hill et al., 2012).

With this study, we believe to have provided, at least in part, an answer to the question as posed by Tedeschi and Di Giovanni: “which are the non-apoptotic p53 transcriptional targets in developing and in mature neurons following injury?” (Tedeschi and Di Giovanni, 2009). We have identified several new p53 targets with potentially important functions in (DNA damage-induced) neuronal death or differentiation, which may ultimately lead to a better understanding of the different processes involved in nervous system development. However, many questions about the exact function of some of these genes still remain unanswered, warranting their further investigation.

## Supplementary Material

Supplementary Material
