# Supplementary material for: Identification of novel radiation-induced p53-dependent transcripts extensively regulated during mouse brain development
Source: Biol Open. 2015 Feb 13;4(3):331–44. doi: 10.1242/bio.20149969 (PMC4359739; doi:10.1242/bio.20149969)
Supplement: Supplementary Material [file supp_4_3_331__index.html]

Identification of novel radiation-induced p53-dependent transcripts extensively regulated during mouse brain development — Supplementary Material 

# Identification of novel radiation-induced p53-dependent transcripts extensively regulated during mouse brain development

## bio.20149969 Supplementary Material

**Files in this Data Supplement:**

- Supplementary Material - Roel Quintens et al. doi: 10.1242/bio.20149969
- Table S3 - **Radiation-responsive gene signature.**
