# Supplementary material for: Identification of novel radiation-induced p53-dependent transcripts extensively regulated during mouse brain development
Source: Biol Open. 2015 Feb 13;4(3):331–44. doi: 10.1242/bio.20149969 (PMC4359739; doi:10.1242/bio.20149969)
Supplement: Supplementary Material [file supp_bio.20149969_bio.20149969-s1.pdf]

Biology Open (2015) 000, 1–14 doi:10.1242/bio.20149969

Supplementary Material
Roel Quintens et al. doi: 10.1242/bio.20149969

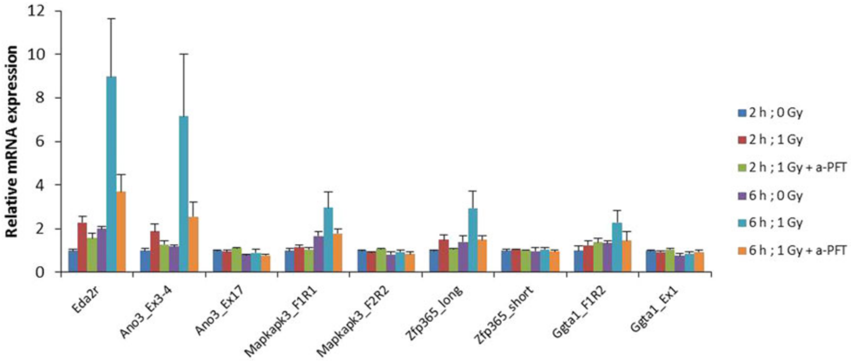

Fig. S1. p53-target gene expression in immature (1 DIV) primary neuronal cultures treated with the p53 inhibitor α-PFT. mRNA expression was determined by qRT-PCR in primary neuronal cultures (1 DIV) at 2 h and 6 h post-irradiation (n=4). The p53 inhibitor a-PFT was added to the medium 90 min prior to the irradiation. Transcript-specific primers (cf. supplementary material Table S1) were used to show differences in expression profiles between specific transcript variants. Error bars represent s.e.m.

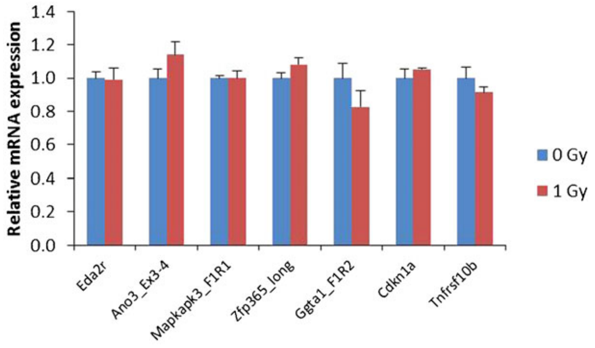

Fig. S2. p53-target gene expression in 7 DIV primary astroglial cultures. mRNA expression was determined by qRT-PCR in primary astroglial cultures at 6 h post-irradiation (n=3). Error bars represent s.e.m.

Biology Open (2015) 000, 1–14 doi:10.1242/bio.20149969

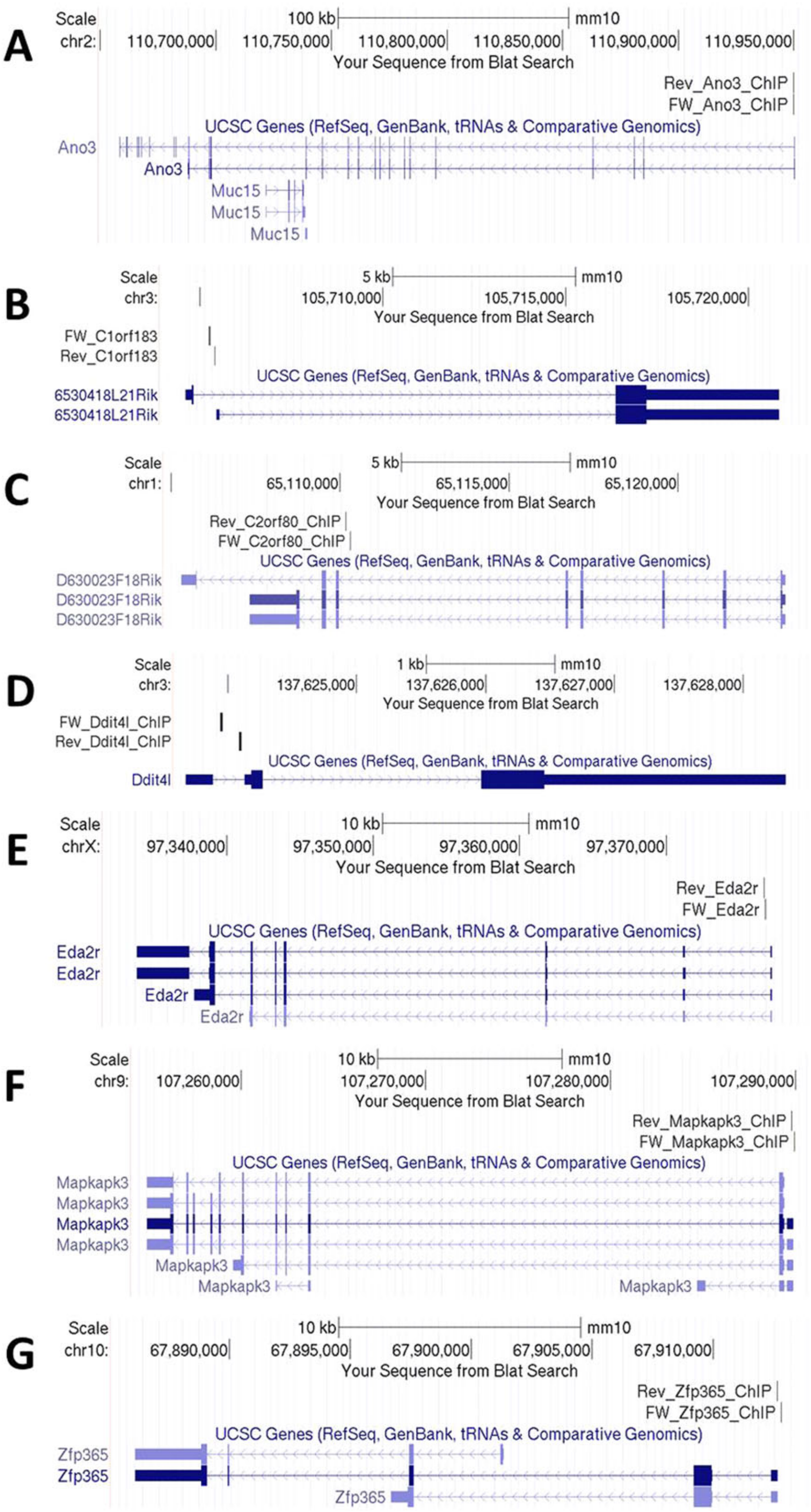

Fig. S3. Genomic positions of PCR primers used for ChIP-PCR (cf. supplementary material Table S2). (A) Ano3, (B) C1orf183, (C) C2orf80, (D) Ddit4l, (E) Eda2r, (F) Mapkapk3, (G) Zfp365. For each panel, the upper track indicates the positions of the primers whereas the lower track represents the known transcript variants. Arrows in introns indicate the orientation of the gene. Note that for *C2orf80* (C), the amplified sequence is just upstream of the alternative transcription start site of the putative short transcript variant.

Biology Open (2015) 000, 1–14 doi:10.1242/bio.20149969

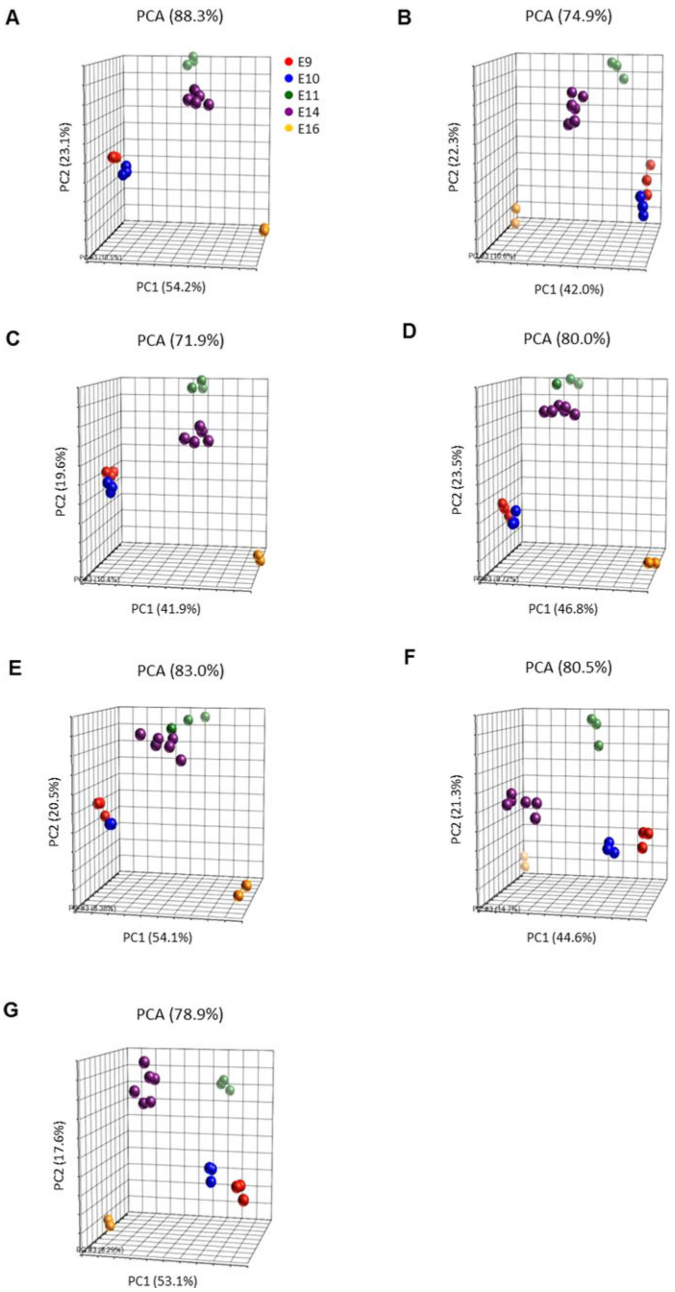

Fig. S4. Principal component analysis of different gene sets during early embryonic brain development. (A) All significant genes, (B) DEX gene signature, (C) apoptotic genes, (D) cell cycle genes, (E) DNA repair genes, (F) brain development genes and (G) neuron differentiation genes.

Biology Open

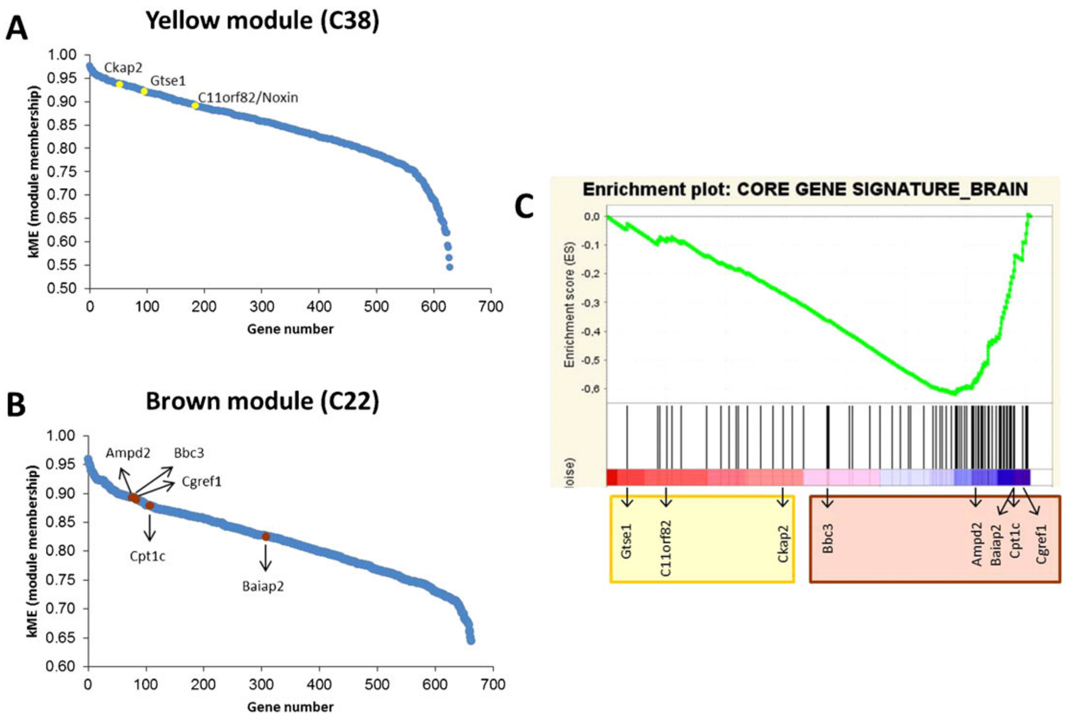

Fig. S5. Radiation-induced DEX genes are also regulated during human embryonic brain development. (A,B) Genes are sorted according to their module membership value (kME). Hub genes for each module are genes with the highest kME. Data taken from publically available results (from Miller et al., 2014). (C) GSEA enrichment plot as shown in Fig. 6B. Members of yellow and brown gene modules are indicated.

Table S1. Primer sequences for qRT-PCR

Gene name	Forward primer (5′→3′)	Reverse primer (5′→3′)
Ano3_Ex17	CTTGGTCATCACAGCAGTGTTC	AGACAGCAGCACCAGATGTAG
**Ano3_Ex3-4**	**AAGGACTCTACCCTTAAGTGTTCC**	**GGAGACGAGATCGATCATAGTTGG**
**C1orf183**	**GAGAGCAAGGACATGGACTG**	**TAGGAGCTTCAGTTCCTGAAGT**
C2orf80_long	CCATCAGTGTTGCCAGTCAATG	CGCAGGTCTGACATGAAGTG
**C2orf80_short**	**GAATGGAGATCCTGTCGGGAAAG**	**TCCTCCACATAACAGTTGAGGAGT**
**Eda2r**	**CCTACCTCAGATGGGACATCA**	**CGGTACTCATTCTCTTGACAATCCA**
Gapdh	CCAATGTGTCCGTCGTGGATCT	GTTGAAGTCGCAGGAGACAACC
**Ggta1_F1R2**	**TTCCATCCTCAAGATCTGGATCA**	**CCTCTGGAATTCTGTTGACATATTCC**
Ggta1_Ex1	AGAACAGATCTGACTGCCTCTTTC	GAGTCTACAAGGGAAGGTGTCAG
**Kank3**	**GCTGATGGCAATGGAAACACAG**	**GCAGAGTAGCCAGCTCTATTCTG**
**Mapkapk3_F1R1**	**CGCATCCTGGACGTGTATGAG**	**CAATGTCCCGCATTATCTCTGCA**
Mapkapk3_F2R2	GCCATGTTGCTGTCTGAGAATAC	CCCAGGAAAGCAAGCAATCAG
**Nr1d1**	**GTGACCCTGCTTAAGGCTG**	**CTCCTGCAGACTGTAGGTTGT**
**Pqlc3**	**GTCCTCCTGCTCTTCGTCTTTC**	**CATGGCCAAGTCGATGATCCA**
**Sec14l5_F1R1**	**CAGCTTCTGTGCTCTCTCTG**	**CCATGACCAGCTCGAATGG**
Sec14l5_F2R2	AGGAGAGTTGCCTGGTTCAG	CCTTGTCCAGGTGGAAGTCTC
Trp53	CACAGCGTGGTGGTACCTTA	TCTTCTGTACGGCGGTCTCT
**Usp2**	**GAGTCCCTGAAGTCCTCAGTC**	**AGACCCTGGGCACTCTTTG**
**Zfp365_long**	**CTCACAGACATCCCATCGAACA**	**CTTCTCCCCTTCAGATGGAAATGG**
Zfp365_short	TGGGCAGCAGCAGTCTATG	CGAGTCAAGAGCTTGAAGCTGG

Bold, radiation-responsive gene (variant).

Biology Open (2015) 000, 1–14 doi:10.1242/bio.20149969

Table S2. Primer sequences for ChIP-PCR

Gene name	Forward primer (5′→3′)	Reverse primer (5′→3′)
Ano3	TGCAACGGAGATAAGTCTGATATCC	CTGCAATGCACTGAAGGAGAC
C1orf183	CTGTGCAGGAGGTCAGGCT	CCCGTCATTACAGCTGCCA
C2orf80	GTAGTGTGCATCGGTTTGTGG	CTTTGCACAGGACATTCCTCAC
Cdkn1a	CGGAGACCAGCAGCAAAATCG	TGACACATACACACCCCAGGCAC
Ddit4l	CAGCTACTTCCAGCCTGTTG	GCAAAAGCACGCAAGAAGTG
Eda2r	CGCTGTTATCCCTACAGCTTTG	CCAACAACCCTGTGCATCTG
Mapkapk3	CGCTGCGAAAGTTACAGTGAAC	TCGTCGCGTCATTCAGACTTG
Zfp365	CAGCTCTTTCCAGGGGCTAG	CTCAGGATTCCGCTGACATCAC

Table S3: See supplementary material webpage

Biology Open
