# Supplementary material for: Identification of novel radiation-induced p53-dependent transcripts extensively regulated during mouse brain development
Source: Biol Open. 2015 Feb 13;4(3):331–44. doi: 10.1242/bio.20149969 (PMC4359739; doi:10.1242/bio.20149969)
Supplement: Supplementary Material [file supp_bio.20149969_BiO9969_Table_S3.docx]

**Table S3. Radiation-responsive gene signature**

| Affymetrix Transcript ID | Gene Symbol | Refseq |
| --- | --- | --- |
| 10481272 | 1700007K13Rik | NM_027040 |
| 10565570 | 4632434I11Rik | NM_001080995 |
| 10491329 | 4930429B21Rik | NR_027966 |
| 10396919 | 4933426M11Rik | NM_178682 |
| 10495094 | 6530418L21Rik | NM_175398 |
| 10397763 | 9030617O03Rik | NM_145448 |
| 10554233 | Aen | NM_026531 |
| 10501244 | Ampd2 | NM_028779 |
| 10485718 | Ano3 | NM_001128103 |
| 10485745 | Ano3 | NM_001128103 |
| 10371846 | Apaf1 | NM_001042558 |
| 10530100 | Arap2 | NM_178407 |
| 10398885 | AW555464 | NM_001024602 |
| 10472893 | B230120H23Rik | NM_023057 |
| 10383289 | Baiap2 | NM_130862 |
| 10563303 | Bax | NM_007527 |
| 10550290 | Bbc3 | NM_133234 |
| 10500710 | BC037703 | NM_172295 |
| 10467956 | Bloc1s2 | NM_028607 |
| 10472095 | Bloc1s2 | NM_028607 |
| 10357875 | Btg2 | NM_007570 |
| 10385271 | Ccng1 | NM_009831 |
| 10460841 | Cdc42bpg | NM_001033342 |
| 10443463 | Cdkn1a | NM_007669 |
| 10529034 | Cgref1 | NM_026770 |
| 10577508 | Ckap2 | NM_001004140 |
| 10562989 | Cpt1c | NM_153679 |
| 10397054 | Dcaf4 | NM_001165256 |
| 10393926 | Dcxr | NM_026428 |
| 10496373 | Ddit4l | NM_030143 |
| 10494889 | Dennd2c | NM_177857 |
| 10605874 | Eda2r | NM_001161432 |
| 10592237 | Ei24 | NM_007915 |
| 10360684 | Ephx1 | NM_010145 |
| 10345981 | Ercc5 | NM_011729 |
| 10462603 | Fas | NM_007987 |
| 10368886 | Foxo3 |  |
| 10368888 | Foxo3 | NM_019740 |
| 10482059 | Ggta1 | NM_010283 |
| 10512640 | Gne | NM_015828 |
| 10374998 | Gpr75 | NM_175490 |
| 10517401 | Grhl3 | NM_001013756 |
| 10426016 | Gtse1 | NM_013882 |
| 10542397 | H2afj | NM_177688 |
| 10394778 | Hpcal1 | NM_016677 |
| 10586306 | Igdcc4 | NM_020043 |
| 10444028 | Kank3 | NM_030697 |
| 10399478 | Lpin1 | NM_015763 |
| 10569181 | Lrdd | NM_022654 |
| 10596637 | Mapkapk3 | NM_178907 |
| 10390691 | Nr1d1 | NM_145434 |
| 10350146 | Phlda3 | NM_013750 |
| 10407126 | Plk2 | NM_152804 |
| 10456357 | Pmaip1 | NM_021451 |
| 10411306 | Polk | NM_012048 |
| 10379891 | Ppm1d | NM_016910 |
| 10399540 | Pqlc3 | NM_172574 |
| 10485624 | Prrg4 | NM_178695 |
| 10417065 | Rap2a | NM_029519 |
| 10492335 | Rap2b | NM_028712 |
| 10565852 | Rnf169 | NM_175388 |
| 10516932 | Sesn2 | NM_144907 |
| 10351259 | Slc19a2 | NM_054087 |
| 10444244 | Tap1 | NM_013683 |
| 10354168 | Tbc1d8 | NM_018775 |
| 10549361 | Tm7sf3 | NM_026281 |
| 10372488 | Tmem19 | NM_133683 |
| 10372917 | Tmem5 | NM_153059 |
| 10416230 | Tnfrsf10b | NM_020275 |
| 10533304 | Trafd1 | NM_001163470 |
| 10503259 | Trp53inp1 | NM_021897 |
| 10584634 | Usp2 | NM_198092 |
| 10570771 | Vps36 | NM_027338 |
| 10499612 | Zbtb7b | NM_009565 |
| 10369783 | Zfp365 | NM_178679 |
| 10394119 | Zfp750 | NM_178763 |
| 10497673 | Zmat3 | NM_009517 |
| 10424437 |  | --- |
| **10355205** | **D630023F18Rik** | **BC137870** |
| **10563858** | **Gabrg3** | **NM_008074** |
| **10471139** | **Hmcn2** | **ENSMUST00000074390** |
| **10543017** | **Pdk4** | **NM_013743** |
| **10352661** | **Ptpn14** | **NM_008976** |
| **10424404** | **Pvt1** | **NR_003368** |
| **10393341** | **Rhbdf2** | **NM_172572** |
| **10433373** | **Sec14l5** | **NM_001127725** |
| **10578574** | **Stox2** | **NM_175162** |
| **10389339** | **Usp32** | **NM_001029934** |

Bold, DAS genes.
